# A Prospective Randomized Controlled Trial Comparing Clinical Equivalence of PD Synth and PDS Polydioxanone Sutures

**DOI:** 10.7759/cureus.50293

**Published:** 2023-12-10

**Authors:** Amritha Prabha Shankar, Stanley Mathew, V S S Nagababu Tippana, Keerthi B.R, Saleem Naik, Ravinder K Pandita, Badareesh L, Naveen Kumar AN, Venkata Narasimha Rao V, Bharath Kumar Bhat, Ashok K Moharana, Deepak TS

**Affiliations:** 1 Department of Surgical Oncology, Vydehi Institute of Medical Sciences and Research Centre, Bengaluru, IND; 2 Department of General Surgery, Kasturba Medical College and Kasturba Hospital, Manipal, IND; 3 Department of General Surgery, King George Hospital/Andhra Medical College, Visakhapatnam, IND; 4 Department of Surgical Oncology, Vydehi Institute of Medical Sciences & Research Centre, Bengaluru, IND; 5 Department of General Surgery, Batra Hospital and Medical Research Center, New Delhi, IND; 6 Department of Surgical Oncology, Kasturba Medical College, Manipal, IND; 7 Department of Surgical Oncology, Kasturba Medical College and Kasturba Hospital, Manipal, IND; 8 Department of Gastrosurgery, Kasturba Medical College and Kasturba Hospital, Manipal, IND; 9 Department of Clinical Affairs, Healthium Medtech Limited, Bengaluru, IND

**Keywords:** polydioxanone suture, midline laparotomy, incisional hernia, fascial dehiscence, absorbable suture

## Abstract

Introduction

Incisional hernia is a common complication of midline laparotomy that may develop even after several years of surgery. Abdominal fascia closure with ideal suture material reduces the incidence of incisional hernia. This study compared the clinical equivalence of PD Synth (Healthium Medtech Limited) and PDS (Ethicon, Johnson & Johnson) slowly absorbed polydioxanone suture with respect to the occurrence of incisional hernia, following elective/emergency midline laparotomy.

Methods

Eighty-eight subjects undergoing elective/emergency midline laparotomy were randomized to PD Synth (n=45) and PDS (n=43) groups of this prospective, multicenter, randomized (1:1), single-blind, two-arm, parallel-group study (December 2020-May 2023). Primary endpoint was incidence of incisional hernia, occurring within six and 12 months of surgery. Secondary endpoints included incidence of fascial dehiscence, surgical site infection (SSI), suture sinus, seroma, hematoma, scar tenderness, and re-suturing, and evaluation of operative data, hospital stay, intra-operative suture handling, pain, time to return to normal day-to-day activities and work, overall patient satisfaction score, and adverse events.

Results

One subject in both PD Synth and PDS groups (p>0.05) developed incisional hernia at umbilicus 12 months post-laparotomy. In PDS^ ^group, one subject each had incidences of SSI on day 2, day 7, and one month, two subjects developed seroma on day seven, and one subject had readmission on one month; two subjects in PD Synth group developed superficial SSI (one month). Findings of other secondary endpoints were comparable between the groups.

Conclusion

Primary and secondary outcomes manifested that PD Synth and PDS^ ^slowly absorbed polydioxanone sutures are clinically equivalent, and can be used for abdominal fascial closure following midline laparotomy.

## Introduction

Quick and easy abdominal access prevailed midline laparotomy as the most frequently used technique in the field of emergency/elective surgery [1]. Patients presenting life-threatening clinical conditions undergo laparotomy only after provisional diagnosis [2]. According to the Centers for Disease Control (CDC), approximately four to five million laparotomies are performed per year in the United States alone [3]. However, post-laparotomy development of incisional hernia is common (9.9%), especially after midline abdominal incision, which further causes 2-20% surgical morbidity [4]. Although incisional hernia may develop several years after surgery, 50% of cases are diagnosed within one year [5]. The rate of incidence may vary from 5-20% depending upon follow-up duration, techniques used for closure of the incision, and biological factors, associated with patients healing, which may increase up to 40% in high-risk patients [6]. This affects patient’s quality of life, and around 30% of the hernias need to be repaired, adding financial burden [3,7].

Wound dehiscence is another post-laparotomy complication, 0.2-5% results from elective laparotomy, and up to 45% are associated with emergency laparotomy, leading to 30% incidence of mortality and morbidity [8]. Ideal closure of midline laparotomy incision is crucial to minimize the occurrence of incisional hernia and wound dehiscence, along with other frequently occurring post-operative complications like wound pain and surgical site infection (SSI) [9]. Investigation of best technique (interrupted vs. continuous suture) for wound closure that may result in lesser complications has been controversial for a long time. The best technique must be simple and convenient that can provide tensile strength throughout the process of healing and good approximation of the tissue, with lesser chances of wound infection [10]. The guidelines from the European and American hernia societies, developed using (GRADE) approach have suggested the use of slowly absorbable suture with continuous suture technique for closure of incision in elective midline laparotomy [11]. The use of absorbable suture was reported to reduce wound pain and risk of sinus formation when compared to non-absorbable suture [12,13]. Monofilament suture material lowers the incidence of SSI [12]. Previous studies have compared monofilament absorbable polydioxanone with non-absorbable polypropylene or polyamide nylon suture for abdominal wound closure and reported comparatively lower wound complication rates with the former [14,15]. However, comparative study on two commonly used brands of slowly absorbed polydioxanone sutures for fascia closure in a single layer is not available. Therefore, this study was designed to compare the clinical equivalence of PD Synth (Healthium Medtech Limited) and PDS (Ethicon, Johnson & Johnson) slowly absorbed polydioxanone sutures, for abdominal fascial closure following elective/emergency midline laparotomy.

## Materials and methods

Study design

A multicenter, prospective, two-arm, parallel-group, randomized (1:1), single-blind 12-month follow-up study was conducted between September 2020 and May 2023. The primary objective was to compare the rate of incisional hernia with PD Synth and PDS polydioxanone sutures, at six months and 12 months post-abdominal fascial closure. Secondary objectives included assessment of SSI, fascial dehiscence, suture sinus, overall intra-operative handling, tissue reaction and material problems, post-operative discomfort, pain, overall patient satisfaction score, and other adverse events.

Ethical approval

The study was registered in the Clinical Trial Registry of India and carried out in compliance with the Declaration of Helsinki. Ethical approval was granted by the Ethics Committees of participating sites. The study complied with ICH-GCP E6(R2), EN-ISO 14155:2020, Medical Device Rules India 2017, Medical Devices Regulation (EU) 2017/745, New Drugs and Clinical Trials Rules 2019, and Consolidated Standards of Reporting Trials (CONSORT) guidelines.

Study setting and participants

Surgical departments in four tertiary care centers across India were involved in this study. Female or male adults (18-60 years) with good systemic/mental health (as per opinion of the investigator) and CDC surgical wound classification class I/II/III requiring elective/emergency midline laparotomy were included in this study. Written informed consent was obtained from all participants.

Participants with body mass index (BMI) of <18.5 and ≥30 kg/m^2^, American Society of Anesthesiologists (ASA) class V, undergoing elective/emergency laparoscopic abdominal surgeries, requiring an early (within 30 days) reintervention after index surgery, or prophylactic mesh augmentation after midline laparotomy were excluded from the study. Participants with history of midline laparotomy, or allergy to polydioxanone or similar products, abdominal hernia, or systemic diseases (chronic obstructive pulmonary disease, tuberculosis, and bleeding disorders), or who were pregnant or planning a pregnancy in the next year were excluded. Participants having a life expectancy of <one year, active infection at/around skin incision site, abdominal hernia or wall metastases, or experimental drug or medical device within 30 days prior to the planned start of the procedure were also excluded. Other exclusion criteria were habit of drug abuse, participation in another trial, unlikely to comply with current surgical procedure or complete the study follow-ups (in the opinion of investigator), direct involvement in the proposed study or other studies under the direction of that investigator or study center (employees of the investigator or study center) and other indication-based exclusion.

Intervention

PD Synth suture (Healthium Medtech Limited) and PDS II (Ethicon, Johnson & Johnson) are both sterile, absorbable, synthetic, monofilament, polydioxanone surgical suture, intended for use in approximation of the general soft tissue, including pediatric cardiovascular tissue in micro and ophthalmic surgery. Both sutures are particularly useful where the combination of an absorbable suture with extended wound support for up to six weeks is desirable.

Study procedure

The primary cause for midline laparotomy was corrected, and abdominal fascia was closed in a single layer with wide bites through the rectus sheath (minimum 1 cm from the incision edge) using either of the sutures. For both sutures, a similar strength of material was used and suture to laparotomy wound length ratio was kept at least 4:1. The skin was closed with surgical staples and the surgeon reconfirmed proper closure, leaving little room for surgical error. The primary dressing was removed after 24-48 hours, and further wound care was done as per the institutional protocol. The wound was inspected for signs of infection and dehiscence before each dressing, and subjects having infection were put on antibiotics according to the institutional protocol and culture and sensitivity report. Skin staples were removed in a conventional way in one to three weeks, based on investigator's discretion.

Subjects were screened (month -3 to day -1) and enrolled to undergo elective/emergency midline laparotomy (day 0, baseline visit). Six post-operative visits were conducted on day three, day seven, month one, month three, month six, and month 12 to record the outcomes.

Demographics and other relevant characteristics

At screening, subjects' age, gender, ethnicity, occupation, weight, height, BMI, occupation, and history of alcohol consumption and smoking were recorded. Vital signs including respiratory rate, pulse rate, and systolic and diastolic blood pressure were also measured. In addition, radiation therapy (if done), medical/surgical history, and physical examination for any abnormality (central nervous system, respiratory system, gastrointestinal system, skin, joints and extremities, ear, nose and throat, general appearance, edema, and lymph nodes) were noted. Pre-surgery pain at screening visits using the visual analog scale (VAS) was evaluated.

Study outcomes

Primary Endpoint

Incidence of incisional hernia, occurring within six and 12 months of the primary surgery in both groups was assessed clinically. Ultrasound examination was performed at investigators' discretion to confirm the presence of an incisional hernia and for its complete evaluation.

Secondary Endpoints

Secondary endpoints were incidence of fascial dehiscence, SSI, suture sinus, seroma, hematoma, and scar tenderness. In addition, intra-operative suture handling parameters (ease of passage through tissue, first-throw knot holding, knot tie-down smoothness, knot security, surgical handling, suture fraying were rated on a five-point scale - 1 poor, 2 fair, 3 good, 4 very good, and 5 excellent), operative data, requirement of re-suturing, and length of hospital stay were assessed. Operative data included length of surgery (time from skin incision to completion of skin closure), suture size, needle-tip geometry and diameter, suture length, and wound length ratio, length of the incision, method of suturing, duration of surgery, blood loss, number of sutures used, antibiotic and thrombosis prophylaxis, drain, use of epidural catheter and suture-related challenges. Time to return to normal day-to-day activities and work were also recorded.

Pain was measured using VAS (0-100 scores), self-completed by the respondent. No pain was designated as 0-4, mild pain as 5-44, moderate pain as 45-74, and severe pain as 75-100. Median patient satisfaction score for overall discomfort and EuroQoL five-dimensional three-level (EQ-5D-3L) questionnaire for overall well-being of the subject were evaluated. Five dimensions of subjects' health state, viz., mobility, self-care, usual activities, pain/discomfort, and anxiety/depression were estimated on three following levels: no problems, some problems, and extreme problems. EuroQol-visual analog scales (EQ-VAS), part of EQ-5D were used for global assessment of subjects' health on a vertical VAS (100, best imaginable health and 0, worst imaginable health).

Clinical signs, injury, or disease not reported as the study endpoint was noted as an adverse event (AE). Side effects related to the standard care for index disease, a condition requiring a pre-planned procedure (unless the condition worsened since screening), or a pre-existing condition were not labeled and reported as an AE. Moreover, a serious adverse event (SAE) was defined as AE that led to serious deterioration of health, permanent impairment of body structure or function, re- or prolonged hospitalization, or death. Concomitant or prescribed medications were also listed.

Sample size

The rate of incisional hernia varies greatly between different reports, probably very much related to different definitions of incisional hernia used at follow-up. For sample size calculation, proportion of patients having incisional hernia of 5.6% till 12 months as reported by a previous study was considered in the PDS suture arm [16]. The anticipated proportion of the patients having incisional hernia in the PD Synth suture arm till 12 months was assumed as 6.0%. Considering type I error, power, and margin of non-inferiority as 5%, 80%, and 15%, respectively, requirement of sample size was calculated as 38 in each arm, providing a total sample size of 76. Further, considering 20% dropout and post-randomization exclusion, the required sample size was increased to 92. This sample size was adjusted to 96 for block randomization, with 48 subjects to be enrolled in each arm.

Sample Size Calculation Formula

Two-sample parallel non-inferiority is calculated as follows:

$&pi;_{1} - &pi;_{2} > &delta; n_{i}=\frac{(Z_{&alpha;}+ Z_{&beta;})^{2} (&pi;_{1} (1-&pi;_{2}) + &pi;_{2} (1-&pi;_{2})}{(&pi;_{1} - &pi;_{2} - &delta;)^{2}}$

Here, n_i_ is sample size required in each group, Z_α_ is conventional multiplier for alpha, Z_β_ is conventional multiplier for power, π_1_ is incidence rate of incisional hernia in the standard PDS arm, π_2_ is incidence rate of incisional hernia in PD Synth arm, δ is margin of non-inferiority difference, and π_1_-π_2_ is size of difference of clinical importance.

Randomization and blinding

Block randomization with variable block length, stratified per trial site was performed using a sequentially numbered opaque sealed envelope technique. Eligible subjects were randomized in a 1:1 ratio to receive either of the sutures. An interactive, automated randomization number was generated before the initiation of the study by an independent programmer (not a member of the study team).

Post-operative controls were performed at each center by a physician or support staff, not directly involved in the study, and they were kept blind to the particular suture used in each patient, along with the patient himself/herself. However, the operating staff cannot be blinded to allocation due to the nature of the intervention; they were instructed not to disclose the allocation status at any time point.

Statistical analysis

Per-protocol or PP analysis set was used for subjects, who had complete data for primary endpoint at 12 months follow-up, without major protocol deviations that could impact the primary outcome. The t-test and its non-parametric equivalent (Mann-Whitney U test) were used for comparing mean±SD for all continuous variables. Frequencies and percentages of all qualitative variables were calculated by applying the chi-square test. Data of primary endpoint was expressed as proportion/percentage of subjects having incisional hernia and compared using the chi-square test. Depending on quantitative or qualitative nature of the variables, secondary endpoints were expressed as mean±SD or as proportions/percentages. Data were analyzed using SPSS version 28.0 (Chicago, IL: SPSS Inc.), considering p<0.05 as significant.

## Results

**Figure 1:**
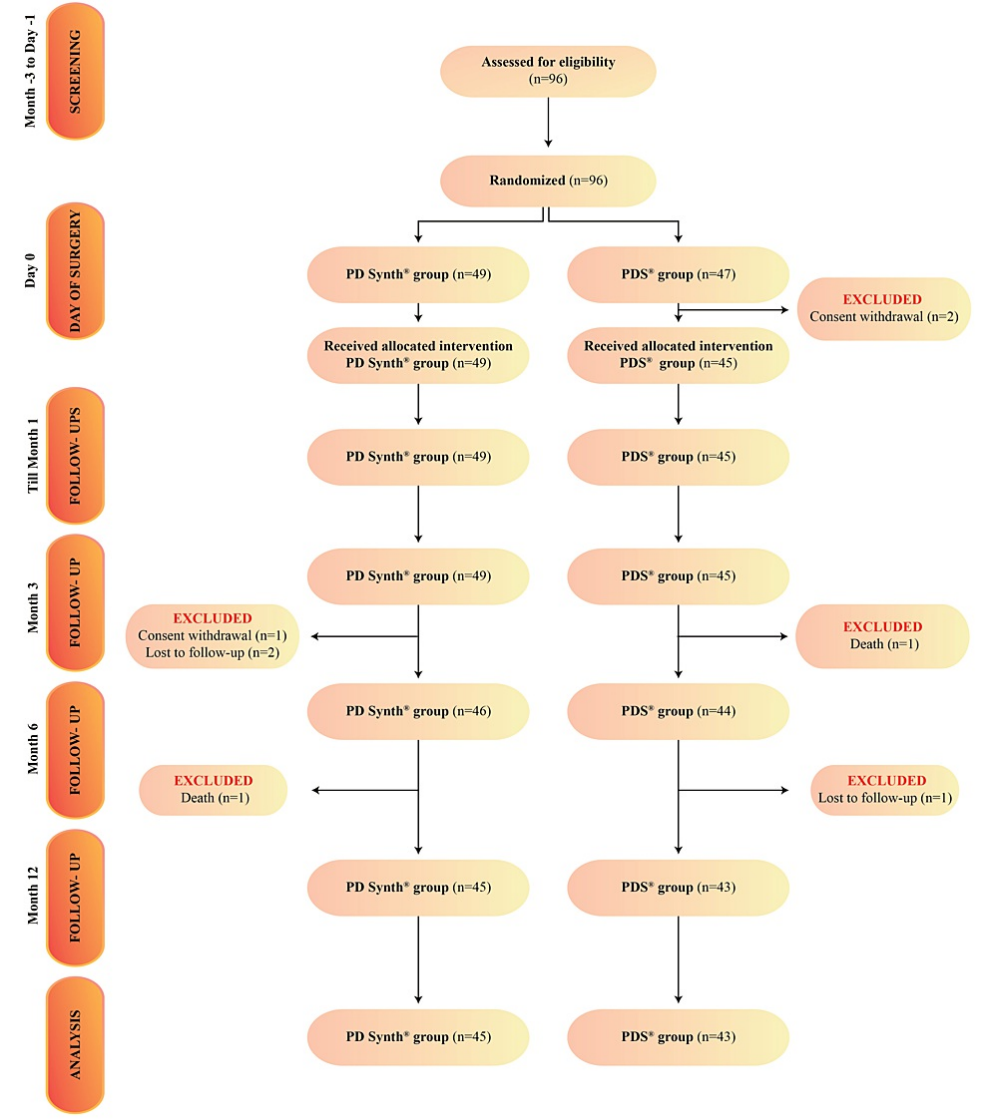
CONSORT flow chart of the study. CONSORT: Consolidated Standards of Reporting Trials n: number of patients

Between December 2020 and June 2022, a total of 96 participants were screened for eligibility. Final analysis included 88 subjects, randomized in PD Synth (n=45) and PDS (n=43) groups, who have completed the trial. The reason for exclusion of the rest of the subjects is presented in Figure 1.

Demographics and other relevant characteristics

Demographics of subjects in terms of age, ethnicity, gender, occupation, alcohol consumption, and smoking history were comparable. All subjects of PDS group were Indians; 44 (97.8%) subjects in PD Synth group were Indians and one (2.2%) subject was non-Indian Asian (p=0.33). In PD Synth and PDS groups, 22 (48.9%) and 20 (46.5%) subjects, respectively, were females and the remaining were males (p=0.82). Both groups were comparable with respect to occupation, vital signs, alcohol and smoking history, and medical/surgical history (Table 1).

**Table 1:** Baseline characteristics of the study participants. Data is presented as mean±SD or n (%). n=number of patients

Subject characteristics	PD Synth (n=45)	PDS (n=43)	p-Value
Age (years)	41.3±12.0	43.6±10.8	0.12
Alcohol consumption history	6 (13.3)	7 (16.3)	0.70
Smoking history	5 (11.1)	2 (4.7)	0.26
Medical/surgical history	38 (84.4)	36 (83.7)	0.99
Weight (kg)	60.0±10.4	59.1±9.5	0.69
Height (cm)	161.2±8.9	159.3±7.7	0.48
BMI (kg/m^2^)	23.0±3.1	23.22±2.8	0.71
Occupation			
Desk job	3 (6.7)	0	0.62
Hard strenuous job	9 (20.0)	9 (20.9)	
Mild strenuous job	17 (37.8)	16 (37.2)	
Housewife	16 (35.6)	18 (41.9)	
Vital signs			
Pulse rate (beats per minute)	84.3±9.7	85.8±7.7	0.22
Respiratory rate (respiration per minute)	18.4±2.3	18.1±2.3	0.79
Systolic blood pressure (mmHg)	118.0±11.1	121.6±10.1	0.62
Diastolic blood pressure (mmHg)	75.2±8.8	77.9±6.9	0.11

Pre-surgery radiation therapy was required in one subject of both PD Synth (2.2%) and PDS (2.3%) groups (p=0.97). Physical examination revealed abnormal gastrointestinal system (100.0% vs. 97.7%, p=0.30), skin (2.2% vs. 4.7%, p=0.53), joint and extremities (0 vs. 2.3%, p=0.30) and lymph nodes (2.2% vs. 4.7%, p=0.53) in PD Synth and PDS groups.

Primary endpoint analysis

Post-operative incidence of incisional hernia was evaluated at month one, six, and 12 follow-ups. There was no incidence of incisional hernia at month one among the subjects of both groups. However, at six and 12-month follow-ups, one (2.2%) subject of PD Synth group was diagnosed with incisional hernia at umbilicus. Ultrasound examination showed that the subject had intact linea alba, but bulging at umbilicus with and without the Valsalva maneuver at both visits. At month six, the size of the defect was 6 mm which increased to 10 mm at month 12 follow-up, and omentum fat was present in the defect. At the last follow-up, presence of incisional hernia at umbilicus was marked in one (2.3%) subject of PDS group, who had focal defect at umbilical region. The subject had bulging with Valsalva maneuver along with a defect measuring 1.7 cm. Also, fatty tissue was present in the defect. The result was comparable between the groups at month six (p=0.96) and month 12 (p=0.97) follow-ups.

Secondary endpoint analysis

Intra-operative Profile

**Figure 2:**
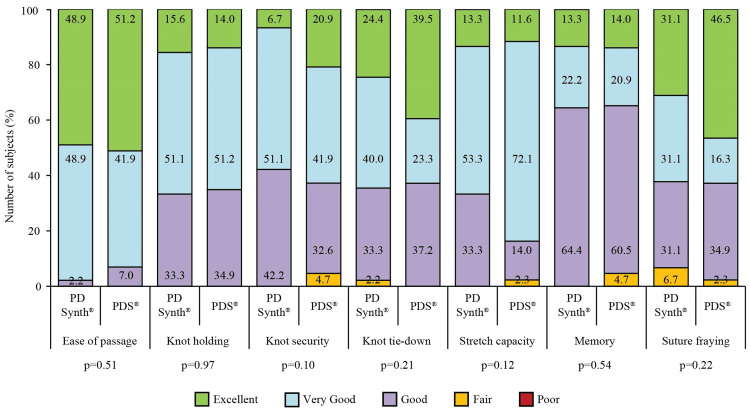
Intra-operative suture handling characteristics in subjects assigned to PD Synth (n=45) and PDS (n=43) groups.

Intra-operative antibiotic prophylaxis was given to all study participants. General anesthesia was used in 42 (93.3%) and 41 (95.3%) subjects of PD Synth and PDS groups, respectively, the rest were given spinal anesthesia (p=0.69). Intra-operative suture handling characteristics were comparable for both suture groups. None of the characteristics were graded as “poor” and no intra-operative suture-related challenge was reported (Figure 2). Continuous suturing with size no. 1 suture of 150.00 cm was done in all subjects of both groups. Round-bodied needles of 50 mm in PD Synth group and 48 mm in PDS group were used. The suture and wound length ratio was 4:1 in both groups. Thrombosis prophylaxis, deep vein thrombosis pump (2.2% vs. 2.3%), Clexane (37.8% vs. 41.9%), and heparin (0 vs. 2.3%) were used in both PD Synth and PDS groups (p=0.37). During the surgery, blood loss occurred in 28 (62.2%) and 26 (60.5%) subjects of PD Synth and PDS groups, respectively (p=0.34). Epidural catheter was used in 13 (28.9%) subjects of PD Synth and 15 (34.9%) subjects of PDS groups (p=0.55). None of the subjects reported perioperative complications (p=1.00). Good outcome of surgery was noted in both groups (p=1.00). Other intra-operative details are summarized in Table 2.

**Table 2:** Intra-operative and post-operative characteristics of the study participants. *Number of subjects, n=28. **Number of subjects, n=26. Data is presented as mean±SD or n (%).

Subject profile	PD Synth (n=45)	PDS (n=43)	p-Value
Intra-operative profile			
Length of incision (cm)	15.5±3.1	15.7±3.4	0.27
Number of sutures used	1.6±0.7	1.7±0.5	0.70
Total operative time (h)	3.0±2.6	3.2±2.1	0.41
Blood loss amount (mL)	331.6±229.6^*^	524.6±548.6^**^	0.81
Number of sutures used			
1	20 (44.4)	16 (37.2)	0.69
2	23 (51.1)	26 (60.5)	
3	1 (2.2)	1 (2.3)	
4	1 (2.2)	0	
Type of drain administered			
Abdominal	1 (2.2)	0	0.88
Pelvic	3 (6.7)	5 (11.6)	
Subcutaneous	0	1 (2.3)	
Esophagojejunal anastomosis site	1 (2.2)	0	
Duodenal stump	0	1 (2.3)	
Morrisons pouch	1 (2.2)	0	
Left hypochondrium	1 (2.2)	0	
Left splenic bled	1 (2.2)	0	
Left flank	0	1 (2.3)	
Flat drain	1 (2.2)	0	
Ryles tube	1 (2.2)	0	
Post-operative profile			
Length of ICU stay (days)	0.6±0.8	0.6±0.8	0.87
Length of hospital stay (days)	9.1±7.8	8.8±6.2	0.86
Time taken to return to normal day-to-day activities (days)	19.4±9.0	17.3±9.8	0.21
Time taken to return to work (days)	38.3±16.3	35.6±18.5	0.54

Post-operative Profile

**Figure 3:**
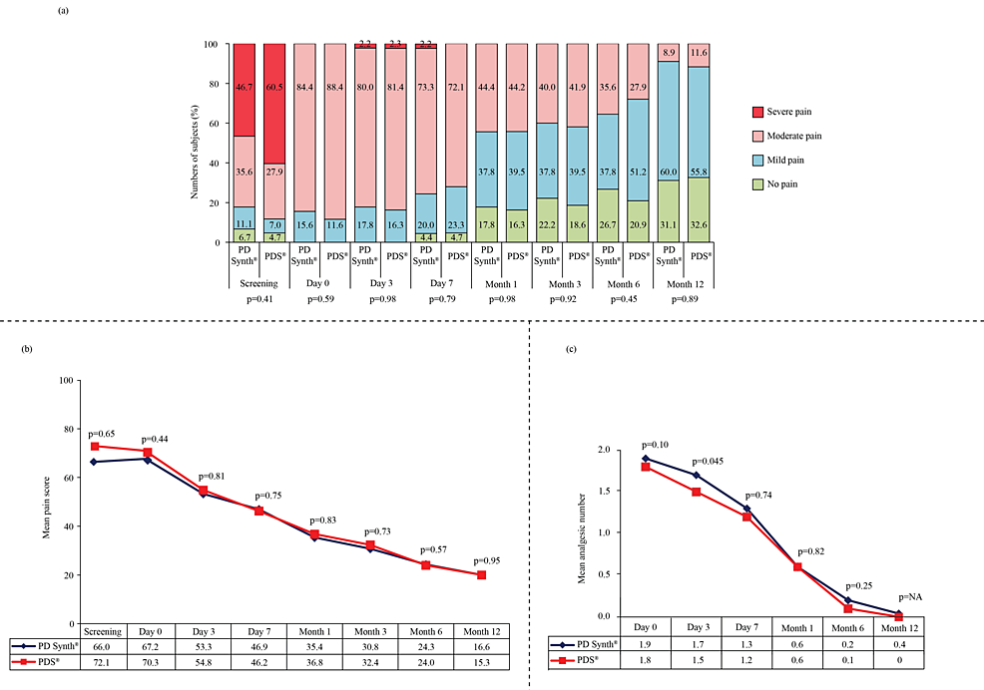
Mean pain score with VAS (a), grade of pain (b), and number of analgesics in subjects assigned to PD Synth (n=45) and PDS (n=43) groups (c). *P<0.05 is considered significant. VAS: visual analog scale

All subjects were screened for fascial dehiscence on day three and seven, and for suture sinus at month one, six, and seven, and no occurrence of the same were noted. However, in PDS group, one (2.3%) subject developed superficial incisional SSI on day two (p=0.99) and day seven (p=0.99). In both PD Synth and PDS groups, two (4.4%) and one (2.3%) subjects, respectively, had superficial incisional SSI at month one, the finding is non-significant (p=0.58). Two (4.7%) subjects of PDS group had seroma on day seven (p=0.98). However, no medications were prescribed for the complications and no further incidence of superficial incisional SSI and seroma were recorded on the subsequent visits. Other post-operative complications, viz., deep incisional, hematoma, scar tenderness, re-suturing, and other suture-related complications did not occur in any subjects of both groups. Pain started at 4.3±2.0 and 4.3±2.7 hours of surgery in subjects randomized to PD Synth and PDS groups, respectively (p=0.65). On days 0, three, and seven, all subjects of PD Synth and PDS groups had pain. However, proportion of subjects, experiencing no pain increased with time, and by month 12, none of them had severe pain (Figure 3, panel a).

Improvement in intra-group pain with each follow-up was apparent in both groups (Figure 3, panel b). In addition, requirement for analgesics was declined with each passing visit, and at month three, only three (6.7%) subjects in PD Synth group and three (7.0%) subjects in PDS group were taking analgesics (p=0.95) (Figure 3, panel c). None of the subjects in both groups reported having complications on month three follow-up, viz., peri incisional swelling, infection, fever, back pain, abdominal colic, vomiting, constipation, distension of abdomen, and difficulty in respiration. At the discretion of the investigator, in PD Synth and PDS groups ultrasound was performed in one (2.2%) and four (9.3%) subjects, respectively, at month six (p=0.15), and in eight (17.8%) and seven (16.3%) subjects respectively at month 12 (p=0.85) follow-ups. Among them, presence of incisional hernia was only confirmed in one subject of PD Synth group (at both month six and 12) and in one subject of PDS group (at only month 12), details of which were provided earlier. In addition, non-intact linea alba was noted in one subject of PD Synth group at month 12 and in one subject of PDS group at both months six and 12, though incisional hernia was not diagnosed in these subjects. Similar findings of intensive care unit (ICU) stay, hospital stay, and time to return to normal day-to-day activities and work were recorded (Table 2).

**Figure 4:**
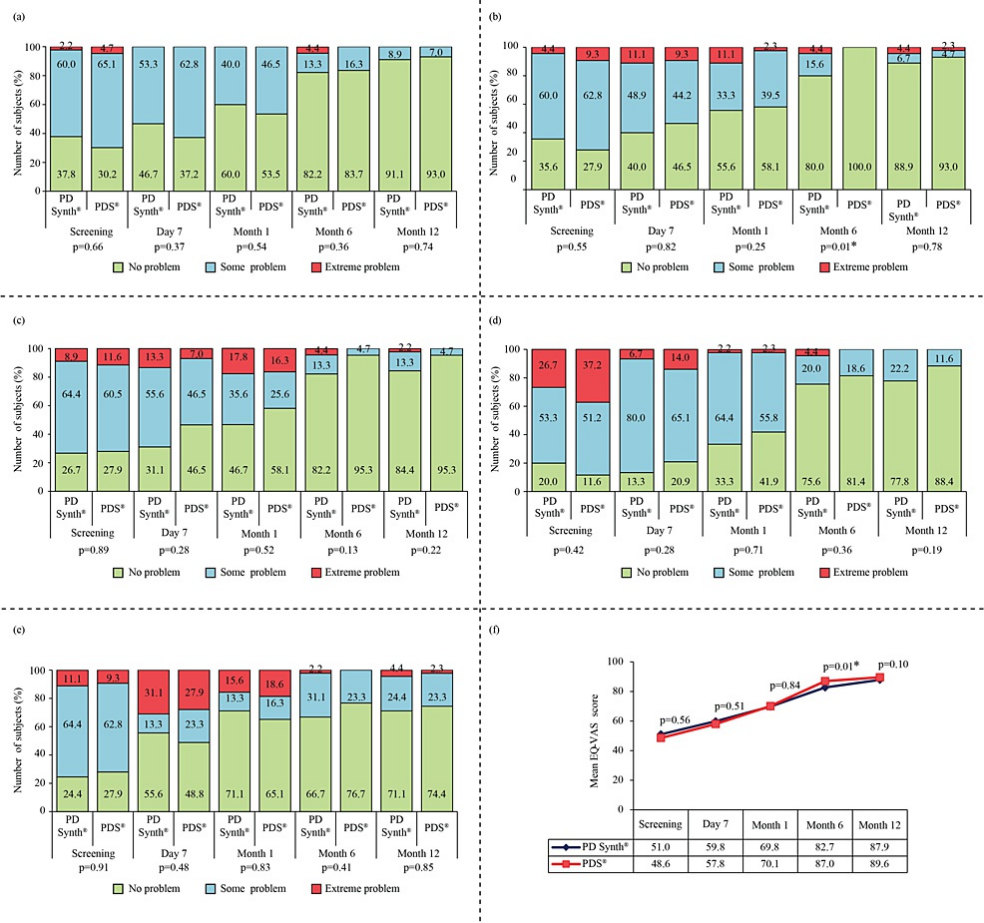
EuroQoL five-dimensional three-level questionnaire for overall well-being. *P<0.05 is considered significant. The images show (a) mobility, (b) self-care, (c) usual activities, (d) pain/discomfort, (e) depression/anxiety, and (f) EuroQol-visual analog scale for global assessment of health in subjects assigned to PD Synth (n=45) and PDS (n=43) groups.

The subjects of both groups reported having some problems in mobility, self-care, usual activities, pain/discomfort, and depression/anxiety at screening visits that improved after undergoing midline laparotomy. Analysis of each dimension of EQ-5D showed that overall proportion of no problems was increased with each post-operative follow-up in both PD Synth and PDS groups (Figure 4, panels a-e).

Between the groups, a significant (p<0.05) difference in self-care was detected only at month six. However, the results for all dimensions were comparable at final follow-up. Additionally, the EQ-VAS score gradually improved with each post-operative visit (Figure 4, panel f). Although at month six, a significant (p<0.05) difference in EQ-VAS score was found between PD Synth (82.73±18.15) and PDS (86.95±11.82) groups, but the mean result was increased and at month 12, a comparable (87.89±12.02 vs. 89.58±7.60) improvement was noted between the groups.

Adverse events and SAEs, occurring within the course of the study were documented. A total of 12 and 13 non-serious mild AEs were reported in PD Synth and PDS groups, respectively. The incidents were not related to the study device. Vomiting and giddiness (2.2%), constipation (2.2%), fat necrosis (2.2%), resuture of skin (2.2%), headache (4.4%), vomiting (2.2%), burning micturition (2.2%), diarrhea (2.2%), hypertrophic scar (4.4%), and general body pains (2.2%) were reported in PD Synth group. Chest pain (2.3%), skin gaping (2.3%), thrombocytosis (2.3%), wound discharge (2.3%), resuture of skin (4.7%), abdominal pain (2.3%), headache (2.3%), headache and nausea (2.3%), anemia (2.3%), cough, constipation and pain localized to upper abdomen (2.3%), cold (2.3%), and constipation (2.3%) were recorded in PDS group. One (2.3%) subject in PDS group was readmitted due to vomiting and abdominal distension at month one, and discharged after treatment to continue the study. This was reported as SAE and not related to the study device. Other SAEs took place but were not included in the PP analysis set due to unavailability of primary endpoint data. The SAEs were as follows: readmission at month one (due to pleural effusion) and month three (due to left-sided chest pain and dyspnea), death (due to intra-capsular neck of femur fracture after falling) of one subject in PD Synth group, and at month six, death (due to hemorrhagic shock) of one subject in PDS group. Both subjects were excluded from the study (Figure 1). Analgesics, antibiotics, and medications for gastritis were prescribed to the subjects during the study; details of some of them are given in Table 3.

**Table 3:** Concomitant or prescribed medications. Data is presented as n (%). n: number of patients

Prescribed medications	PD Synth (n=45)	PDS (n=43)
Analgesics		
Paracetamol	45 (100.0)	42 (97.7)
Tramadol	28 (62.2)	29 (67.4)
Diclofenac	16 (35.6)	10 (23.3)
Fentanyl	15 (33.3)	12 (27.9)
Antibiotics		
Cefoperazone+sulbactam	18 (40.0)	15 (34.9)
Metronidazole	16 (35.6)	19 (44.2)
Cefuroxime	17 (37.8)	13 (30.2)
Ceftriaxone	7 (15.6)	9 (20.9)
Gastrointestinal		
Pantoprazole	33 (73.3)	39 (90.7)
Ondansetron	15 (33.3)	20 (46.5)

## Discussion

Midline laparotomy offers advantage of exposure and ease of access to several organs but still poses risk of impaired wound healing due to avascular nature of linea alba [17]. The development of incisional hernia is a frequent and common complication of laparotomy, elevating the healthcare burden [18]. An increase in intra-abdominal pressure, abdominal distension, and inadequate healing of a previous incision, which is necessary for providing strength and preventing development of hernia, are the contributing factors to hernia development [9,19]. Moreover, higher incidence of incisional hernia is evident with midline incisions as compared to transverse and paramedian incisions [17,20]. Continuous closure of abdominal fascia after midline laparotomies using slowly absorbable monofilament suture material with a suture length:wound length ratio above 4:1 provides stability and mechanical strength [21]. A significant improvement in outcomes of midline laparotomy was reported with the use of polydioxanone sutures, along with a reduced incidence of incisional hernia compared to polypropylene sutures (30.9% vs. 51.1%) [22]. The present study is the first to compare PD Synth and PDS slowly absorbed polydioxanone sutures for the incidence of incisional hernia, occurring within 12 months of abdominal fascial closure following midline laparotomy. The study sheds light on the efficacy and safety of both sutures for elective/emergency midline laparotomy.

Both PD Synth and PDS sutures had satisfactory handling properties regarding ease of passage through tissue, first-throw knot holding, knot tie-down smoothness, knot security, surgical handling, and suture fraying. None of the participants has faced perioperative complications, and as a result, good outcome of surgery was noted in both groups. However, at the end of the study, 2.2% and 2.3% of subjects of PD Synth and PDS groups, respectively, appeared to have incisional hernia at umbilicus. Time-dependent development of incisional hernias has been reported by previous studies; 7.7% incidence within two years [12], 12.8% within 23.7 months (~2 years) [23], and 5% requirement of incisional hernia repair within five years of midline laparotomy [24]. A recent study recorded a 54% incidence of incisional hernia, mostly in the infraumbilical region of Indian patients within three years of midline incision [18]. Reduction of incisional hernia by 5% is associated with a cost saving of four million Euros in French public hospitals [25]. A relatively lower incidence of incisional hernia in the present study contrary to previous findings regarded to reduce the economic as well as healthcare burden of the patients.

Patients undergoing elective laparotomy usually have adequate nutritional status and fewer chances to develop dehiscence because of lower risk factors, compared to emergency patients with multiple risk factors [26]. Incidence of post-laparotomy wound dehiscence is reported as 0.2-5% in elective surgeries and 45% in emergency surgeries; in developing countries, the rate is 30% after undergoing laparotomy for various reasons [8]. A prospective cohort study recorded 12.4% incidence of burst abdomen or wound dehiscence after emergency midline laparotomy [27]. A previous randomized controlled trial observed 12.5% cases of wound dehiscence within one month of emergency midline laparotomy. The authors also noted a significantly higher frequency of abdominal wound dehiscence using interrupted suture technique (20.5%) than continuous suture technique (4.5%) [28]. Similarly, Chalya et al., found lower incidence of wound dehiscence with continuous suturing than with interrupted technique (5.4% vs. 22.1%) and with absorbable suture than non-absorbable suture (7.6% vs. 9.3%) [12]. On the other hand, Sharma et al., favored the use of interrupted suture over continuous suture technique, as 7.9% rate of wound dehiscence/burst abdomen was found with the former technique in comparison to continuous suturing of rectus sheath, which resulted in 19.5% incidence of wound dehiscence after the seventh day of midline incision [29]. In addition, higher occurrence of fascial dehiscence was evident in a retrospective observational study after midline incision (8.1%), compared to transverse incision (3.6%) for elective abdominal surgery [30]. In the present study, patients requiring laparotomy through midline incision in both elective and emergency settings were included, and post-laparotomy abdominal fascia closure was accomplished using PD Synth and PDS polydioxanone sutures in continuous manner. Contrary to the above-mentioned studies, subjects of this study did not develop early post-operative fascial dehiscence within seven days of the surgery.

Other post-operative complications that were noted within one month of midline laparotomy were superficial incisional SSI (in both PD Synth and PDS groups) and seroma (only in PDS group). Incidence of wound infection and seroma after midline laparotomy were found across many previous studies. A study from Central India reported wound infection in 7/60 patients and seroma in 4/60 patients, who underwent elective/emergency midline laparotomy [1]. A recent retrospective cohort study recorded 16.3% and 3.0% incidence of wound infection and seroma, respectively, in adults undergoing midline emergency laparotomy [31]. Clinically 16.7% of patients developed seroma following abdominal surgery through midline incision [32]. Overall 27.9% incidence of wound infection after midline laparotomy in one study [29] and 41.9% in another study were reported [12]. Hempel et al. observed a significant difference in occurrence of SSI after elective abdominal surgery between midline and transverse incision (27.6% vs. 16.8%) [30]. However, with the use of polydioxanone sutures, a significantly reduced SSI was registered compared to non-absorbable polypropylene sutures (23.2% vs. 45.5%) [14].

Post-operative medical and operation-related complications lead to prolonged hospital stays and greater mortality, as found after emergency midline laparotomy [31]. Surgical site infection (20%) was indicated as the major cause for re-hospitalization, followed by sub-acute intestinal obstruction, gastrointestinal causes, burst abdomen, stoma-related, and other causes [33]. Another study also recorded 17.4% unplanned readmission after laparotomy, the most common causes were viscus perforation and small bowel obstruction [34]. However, readmission was noted in only one subject in PDS group of the current study, because of vomiting and abdominal distension, which had no marked impact as the subject completed the next follow-ups successfully. The readmission was not related to the device and was reported as SAE. Hospital stay was ~9 days in both PD Synth and PDS groups, which is comparable to some previous studies that demonstrated post-laparotomy hospital stay of ~10 days [35] and 11-12 days [26].

Chronic persistent post-surgical pain following laparotomy was demonstrated at post-operative day 90 in 38.1% of patients (all had moderate pain) undergoing staging laparotomy that impacted patient’s quality of life [36]. In our study, by the third month, 40.0% and 41.9% of subjects in PD Synth and PDS groups, respectively, had moderate pain, and only three subjects in each group required analgesics. However, improvement in pain and number of analgesics is clearly visible, and at the end of the study 8.9% and 11.6% of patients had moderate pain, while the rest of the patients experienced mild or no pain. Quality of life has also improved, as demonstrated by the results of EQ-5D-5L. No problem in mobility, self-care, usual activities, pain/discomfort, and anxiety/depression was witnessed in the majority of subjects of both groups after one year of surgery. Also, a higher EQ-VAS score of 87.9 and 89.6 in PD Synth and PDS groups, respectively, were recorded at the end of the study. A similar EQ-5D score (80.4±16.7) was reported by Fortelny et al. after one year of fascial closure with absorbent elastic suture material [37]. Furthermore, subjects of both arms have returned to normal day-to-day activities as well as work at a similar time, indicating favorable outcomes of the surgery.

Although the study subjects were blinded to the suture material that was used, hospital staff and practitioners were not blinded due to the nature of the intervention. Hence, there is a probability of potential bias in the reporting if practitioners favor one suture or another. Nonetheless, comprehensive, systematic, and explicit design of the study and careful consideration of inclusion/exclusion criteria are strengths of the study. Comparable outcomes regarding both sutures indicated the use of PD Synth slowly absorbed polydioxanone suture for all surgeries, indicated for PDS slowly absorbed polydioxanone suture.

## Conclusions

To conclude, incisional hernia is a common outcome of elective/emergency midline laparotomy, and our study is no exception. However, the frequency of the incidence is not too many and is non-significant between the studied groups. In addition, at the end of 12 months, results of secondary outcomes of the study showed non-significant differences between the groups. The findings manifested that PD Synth and PDS slowly absorbed polydioxanone sutures are clinically equivalent. Therefore, both PD Synth and PDS sutures can be used in subjects requiring abdominal fascial closure following elective or emergency midline laparotomy.
